# New materials for Li-ion batteries: synthesis and spectroscopic characterization of Li_2_(FeMnCo)SiO_4_ cathode materials

**DOI:** 10.1038/srep27896

**Published:** 2016-06-13

**Authors:** Stefania Ferrari, Maria Cristina Mozzati, Marco Lantieri, Gabriele Spina, Doretta Capsoni, Marcella Bini

**Affiliations:** 1 WMG, University of Warwick, Gibbet Hill Road, Coventry CV4 7AL, UK; 2 Dept. of Physics and CNISM, University of Pavia, via Bassi 6, 27100 Pavia, Italy; 3 Istituto dei Sistemi Complessi – CNR, Via Madonna del Piano 10, 50019 Sesto Fiorentino (FI), Italy; 4 Dept. of Physics and Astronomy, University of Florence, Via Sansone 1, 50019 Sesto Fiorentino (FI), Italy; 5 Dept. of Chemistry, University of Pavia, viale Taramelli 16, 27100 Pavia, Italy

## Abstract

Improving cathode materials is mandatory for next-generation Li-ion batteries. Exploring polyanion compounds with high theoretical capacity such as the lithium metal orthosilicates, Li_2_MSiO_4_ is of great importance. In particular, mixed silicates represent an advancement with practical applications. Here we present results on a rapid solid state synthesis of mixed Li_2_(FeMnCo)SiO_4_ samples in a wide compositional range. The solid solution in the *P2*_*1*_*/n* space group was found to be stable for high iron concentration or for a cobalt content up to about 0.3 atom per formula unit. Other compositions led to a mixture of polymorphs, namely *Pmn2*_*1*_ and *Pbn2*_*1*_. All the samples contained a variable amount of Fe^3+^ ions that was quantified by Mössbauer spectroscopy and confirmed by the T_N_ values of the paramagnetic to antiferromagnetic transition. Preliminary characterization by cyclic voltammetry revealed the effect of Fe^3+^ on the electrochemical response. Further work is required to determine the impact of these electrode materials on lithium batteries.

Promising cathode materials for lithium ions batteries have recently emerged belonging to the Li_2_MSiO_4_ (M = Fe, Mn, Co) orthosilicates family^1^,^2^,^3^. These compounds have attracted great interest due to their high safety and, especially for those based on Fe and Mn ions, also for their low cost, low toxicity and environmental friendliness^4^,^5^. In addition, they all appear particularly interesting due to the theoretical possibility to reversibly de-intercalate two lithium equivalents from the structure, so increasing the overall electrode capacity. In fact, Li_2_MnSiO_4_ could in theory deliver 333 mAhg^−1^, Li_2_CoSiO_4_ 325 mAhg^−1^, while Li_2_FeSiO_4_ 166 mAhg^−1^ for the extraction of one Li ion per formula unit^6^. However, the low electronic conductivity of silicates has to be overcome in order to reach the theoretical capacity: different approaches have been tried, e.g. the doping with Cr, V, Mg, Zn, Cu and Ni^7^,^8^,^9^,^10^, the carbon-coating^11^ or the preparation of composites with carbon nanotubes^12^.

Another critical feature of the orthosilicates, also reported as tetrahedral structures, is their rich polymorphism with numerous crystal structures that, having similar lattice energies, can be stabilized depending on subtle differences in the synthesis conditions^6^,^13^,^14^. Usually, the monoclinic *P2*_*1*_*/n* and the orthorhombic *Pmn2*_*1*_ or *Pmnb* space groups are reported for both the Li_2_MnSiO_4_ and Li_2_FeSiO_4_ compounds^15^,^16^. For Li_2_CoSiO_4_, three stable polymorphs were prepared and structurally characterized: the orthorhombic β_II_ (*Pmn2*_*1*_) and β_I_ (*Pbn2*_*1*_) and the monoclinic γ_0_ (*P2*_*1*_*/n* or *P2/n*)^6^,^17^. The differences among these structures are mainly due to different arrangements of the cation tetrahedra. The polymorphism, with the associated small transition energies, is one of the factors affecting the long-term cyclability of these materials^18^. To gain new insights in this field the relationships between structural and electrochemical properties have been studied by using *in situ* X-ray diffraction measurements during the cell cycling^19^. The formation of either a disordered orthorhombic or monoclinic structure was observed during the lithium extraction, but more studies are needed to better explain the electrochemical behaviour of these compounds.

These issues have recently slightly cooled the initial high interest towards these silicates. To improve the limited structural stability of Li_2_MnSiO_4_ during cycling an interesting approach is represented by the synthesis of mixed Li_2_Fe_1−x_Mn_x_SiO_4_ compounds. As demonstrated by *in situ* diffraction measurements during cell cycling^19^,^20^ and also predicted from DFT calculations^21^, iron ions can have a stabilizing effect when a solid solution of manganese and iron is formed, and more than one electron per transition metal could be delivered in a wide potential range^22^. Intelligent engineering of materials^23^,^24^,^25^ is essential to design the next generation electrode materials, enabling breakthroughs with existing energy and power constraints. A key opportunity is taking advantage of the high capacity and high voltage extraction of Li_2_CoSiO_4_ through a similar methodology as used in developing layered oxides based on multiple transition metals such as LiNi_0.33_Co_0.33_Mn_0.33_O_2_, for which calendar life and safety have been significantly improved^26^. A mixed silicate based on the three Fe, Mn and Co transition metal cations could open a new avenue towards advanced cathodes combining high structural stability, high voltage for the lithium extraction and a competitive cost^27^. In this work we studied a series of Li_2_(FeCoMn)SiO_4_ materials through a combination of X-ray powder diffraction (XRPD), Mössbauer spectroscopy and SQUID magnetometry. At first, we focused on the solid state synthesis of Li_2_Fe_1−x_Co_x/2_Mn_x/2_SiO_4_ (x = 0.2, 0.4, 0.6 and 0.8) to assess the stability of the system and solid solution formation. The results of the structural characterization are herein reported: XRPD with the Rietveld structural refinement was used to study mainly the polymorphism in this compositional range as well as to evaluate the different phases in the samples. The Mössbauer spectroscopy allowed us to determine the valence states of iron in the silicates crystal structures and also to unveil the presence of different iron-containing phases. Finally, the magnetic properties of the compounds were thoroughly investigated by SQUID measurements. A preliminary evaluation of the electrochemical properties by using cyclic voltammetry (CV), is also reported.

## Results

### XRPD and Rietveld results

**Figure 1:**
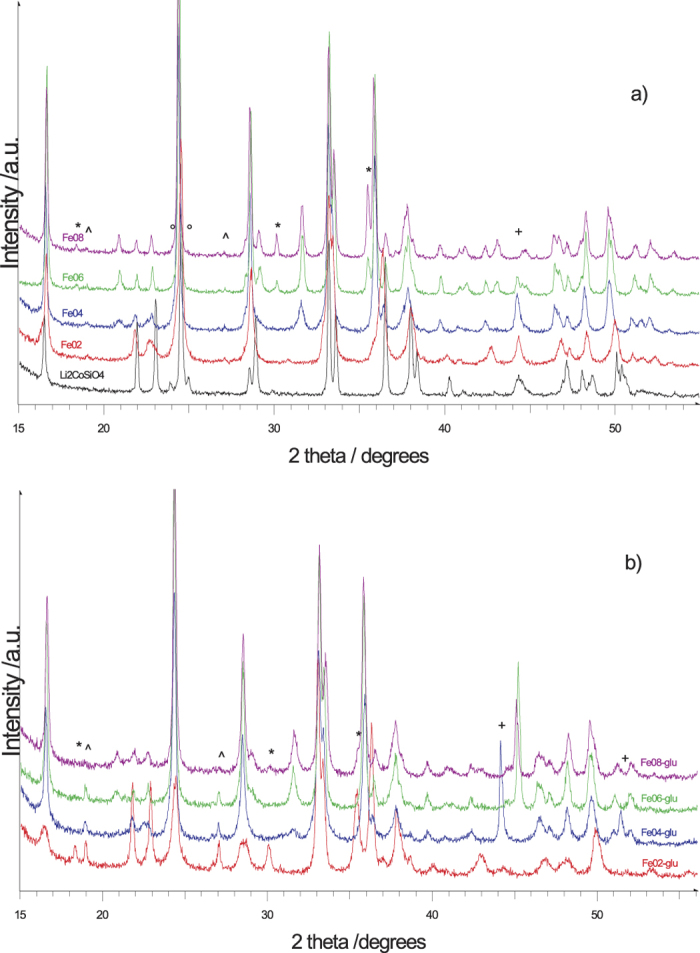
Comparison between XRPD patterns of the samples (**a**) without and (**b**) with glucose addition. The symbols mark the main peaks of magnetite (*), Li_2_SiO_3_ (^), Li_2_Si_2_O_5_ (°) and Co(+).

Figure 1a shows the comparison between the XRPD patterns of carbon-free samples. All the samples are crystalline, although a higher peak broadening is evident for the Fe02 and Fe04 ones. The patterns appear rather different, suggesting the formation of different polymorphs and/or the presence of a small amount of other phases. In Fig. 1b, a comparison between the same samples synthesized with the addition of 6%wt of glucose before the thermal treatment is shown. As expected, a lower degree of crystallinity of these samples with respect to their analogous of Fig. 1a is observed, due to the effect of carbon during the synthesis that is known to prevent particles growth, so causing an evident enlargement of full width at half maximum of peaks. In addition, other peaks appear under 20°/2θ (attributable mainly to lithium silicate impurities) and at about 45°/2θ, where Fe or Co metallic phases have their main reflections. The most noticeable changes still concern the Fe02-glu and Fe04-glu samples.

**Figure 2:**
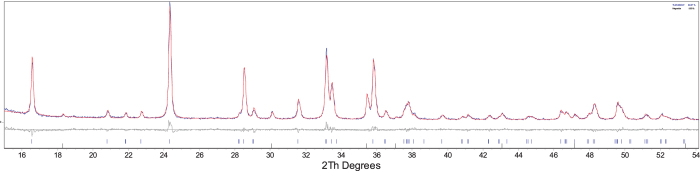
Rietveld refinement of the Fe08 sample pattern. The experimental pattern (blue) is compared with the calculated one (red). The difference plot (gray) and the bars at the expected angular positions of the different phases (orthosilicate s.g. *P2*_*1*_*/n* and magnetite) are also reported.

The Rietveld refinement allowed us to identify the most stable orthosilicate polymorphs for every composition and determine their main structural parameters as well as the kind and amount of the secondary phases. The starting models were the structures reported in the literature for the pure iron, manganese and cobalt silicates, taking into account the existence of different polymorphs^14^,^15^,^28^. The results are shown in Table 1a,b for the pristine and carbon coated samples, respectively. The main structural parameters and the discrepancy factors R_wp_ and S (goodness of fit), are satisfactory thus suggesting a good quality of the structural refinements. In Fig. 2, as an example of the good quality of the Rietveld structural refinements, the comparison between the experimental and calculated pattern together with the difference plot and the reflections bars of the different phases used in the refinement, is reported for the Fe08 sample.

In general, considering the samples with high iron content, that is Fe08, Fe06 and Fe04, the stable polymorph was the monoclinic *P2*_*1*_*/n* one. In the carbon-free samples (Table 1a) the total amount of impurity phases, mainly magnetite and metallic cobalt, was well below 6 wt%, which is an acceptable level of purity. The carbon coated samples (Table 1b) have higher amount of impurities, except for the Fe08-glu one for which a small amount of magnetite and metallic Fe are detected. For all the samples, the lattice volume variation for the orthosilicate phase is negligible for the different compositions suggesting that, the Co and Mn ions substitution mainly involve the iron site, due to the similar ionic radii of Fe, Co and Mn ions^29^. A comment apart is needed for the Fe02 and the Fe02-glu samples (Table 1a,b). In both cases a co-presence of polymorphs in different ratio is observed. For the Fe02 sample, the stable polymorphs are *Pbn2*_*1*_, which is the most stable structure for the pure Li_2_CoSiO_4_ and *Pmn2*_*1*_ typical of the Li_2_FeSiO_4_ compound. Instead, for the Fe02-glu sample a higher amount of the *Pbn2*_*1*_ polymorph is observed together with the *Pmn2*_*1*_ polymorph of Li_2_CoSiO_4_. For the physical characterizations of the carbon coated samples, only the Fe06-glu, will be taken into account and analyzed in detail.

### Mössbauer results

**Figure 3:**
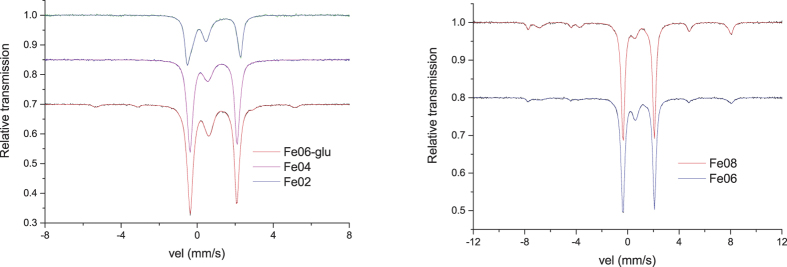
Room temperature Mössbauer spectra collected between ±8 mm/s for Fe06-glu, Fe04 and Fe02 (left side) and between ±12 mm/s for Fe06 and Fe08 (right side).

All the Mössbauer spectra (Fig. 3) are characterized by baseline counts ranging from 3 · 10^5^ to 7 · 10^5^ and show doublet-like components belonging to Fe(II) and Fe(III) sites, differing for quadrupole splitting, isomer shift values and, eventually, magnetic structures rising from iron impurities (e.g. magnetite and FeCo alloy).

The *f*_s_^r^ values for each spectrum were evaluated according to the calibration procedure described in ref. 26, obtaining 0.43 for Fe02, 0.52 for Fe04, 0.56 for Fe06, 0.53 for Fe06-glu and 0.56 for Fe08. The Mössbauer cross-section σ(ω) was expressed by the sum of Voigt sextets on the basis of a standard procedure applied in the case of hyperfine parameters distributions^30^.

In the approximation of anisotropic random orientation of the crystallites in the powder sample and considering that the magnetic interaction, when it is present, is much stronger than the quadrupolar one, the free parameters for each sextet are:

1. The center 〈B_i_〉 and the corresponding standard-deviation σ_*i*_^(*B*)^ of the Gaussian distribution of magnetic fields;
2. The centers 〈δ〉_i_ and 〈Δ〉_i_ with the corresponding standard-deviation σ_*i*_ of the Gaussian distribution of isomer shifts and/or quadrupolar splittings;
3. The contribution *t*_*i*_ to the effective thickness number *t*_a_.

The number of Voigt sextets used to reproduce the Mössbauer line shape depends on the particular sample. The parameter values coming out from the fitting are illustrated for the Fe08 and Fe06-glu samples in the following subsections (for the other samples see the Supplementary Section) and reported in Tables 2 and 3, where we arranged the contributions belonging to the same iron oxidation state by decreasing effective thickness numbers. We underline that for all the samples the parameters values are in agreement with those reported in literature^31^.

Moreover, we stress that σ_*i*_^(*B*)^ and σ_*i*_, which are related to the crystallinity degree (as explained in the next paragraph), are not the FWHM of the spectra lines, which can be immediately estimated by looking at Fig. 3, but they give rise to the anisotropic line broadenings Γ_g_, which appear in the usual expression of the total line width given by^32^:


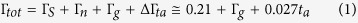


where Γ_s_ and Γ_n_ are the source line width and the natural line width, respectively.

Since the quadrupolar splitting (∆) depends on the distance (*r*) from a point charge as *r*^−3^, it results that δΔ/Δ ≈ 3δr/r. Consequently, by expressing δΔ in terms of the standard-deviation σ_*i*_ (δΔ ≈ 2 √(2 ln 2)σ_*i*_) and considering that two point electronic charges, located at 2.0 Å from the Fe atom, give rise to a quadrupolar splitting of 1.8 mm/s^33^, one finally obtains that the uncertainty of the ligand positions expressed in Å is numerically roughly equal to σ_*i*_. Therefore, in the following we link the crystallinity degree of the samples to the σ_*i*_ values.

### Fe08

**Figure 4:**
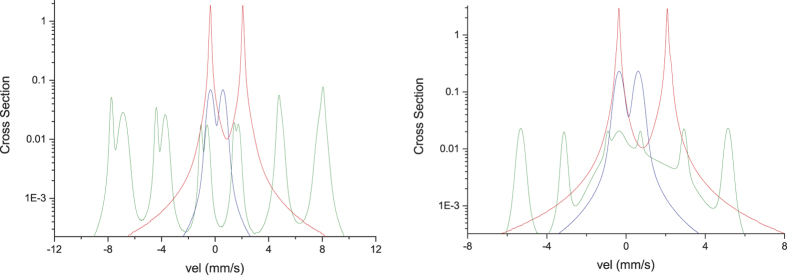
Mössbauer cross section line shapes for the sites of Fe08 (left) and Fe06-glu (right), illustrated in semi-logarithmic scale in order to better display the contributions due to Fe(III) (blue plot) and to impurities (green plot) with respect to the main one belonging to Fe(II) (red plot).

In order to reproduce the experimental line shape, we expressed the Mössbauer cross section by means of six components: three of them belonging to Fe(II) sites, one to Fe(III) and, finally, two to magnetic impurities. In Table 2 we report the parameters of each contribution and in Fig. 4 (left side) we illustrate the cross sections due to Fe(II) sites (78%), Fe(III) site (8%) and impurities sites (14%).

With reference to σ_*i*_ values, the first contribution of Fe(II) can be associated to well crystalline regions in the sample, the second one to regions of medium degree of crystallinity and the last one (with σ_*i*_ ≈ 0.46) to poorly crystalline regions. As far as the contribution of Fe(III) is concerned, it can be connected with medium crystalline regions. The kind of the impurity phase, which is magnetite, was determined on the basis of the hyperfine parameters of the corresponding sites. Its relative amount (*α*) was estimated starting from *t*_*i*_ values. In fact, denoting the mass of the impurity phase with respect to the total mass of the sample by


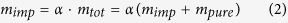


we obtain:


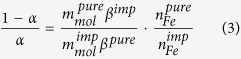


where *β* is the number of iron atoms per stoichiometric formula and *n*_Fe_ is the corresponding number of iron atoms in the sample. Assuming that the Lamb-Mössbauer factor is the same for both the pure and impure phases, finally we have:


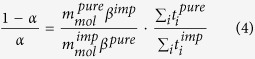


from which we obtained *α* ≈ 0.06, which is actually the same value obtained from XRPD and Rietveld results reported in Table 1a.

### Fe06-glu

The experimental line shape was suitably expressed introducing six contributions to the Mössbauer cross section. Three of them belong to Fe(II) sites, one to Fe(III) and the last two contributions are related to impurities: the former consists of a magnetic hyperfine structure and the latter is used to reproduce a magnetic structure relaxing into a single line. In Table 3 we report the parameters of each contribution and in Fig. 4 (right side) we illustrate the cross sections due to Fe(II) sites (75%), Fe(III) site (19%) and impurity sites (6%). With reference to σ_*i*_ values, the first contribution of Fe(II) can be associated to a well crystalline region, the second and the third ones to medium crystalline regions. The contribution of Fe(III) is connected with a medium crystalline region.

Since the hyperfine field is about 33 T, the impurity phase could be either metal iron or Fe_*x*_Co_1−*x*_ alloys. These alloys, which are consistent with the results of XRPD measurements, show a Mössbauer line shape given by the superposition of a magnetic sub-spectrum and a superparamagnetic one, when the impurity nanograins are smaller than the critical size of ≈ 6 nm^34^,^35^. Analyzing the hyperfine field trend with the relative percentage *x*^36^, a value of 33 T could be associated either to a quasi-pure iron compound (*x* ≥ 0.9) or to a quasi-pure cobalt compound (*x* ≤ 0.2), while for *x* ≈ 0.5 the hyperfine field should be greater than 36 T. Now, assuming in our case *x* ≈ 0.9 (almost pure iron), we would obtain *α* ≈ 0.015 through equation (1) but this result would not be in agreement with the XRPD and Rietveld results (*α* ≈ 0.05). On the contrary, choosing *x* ≈ 0.2 (almost pure cobalt), *α* results to be ≈0.06, which is in good agreement with the XRPD result. We also stress that the presence of small size nanograins (≤6 nm) could be reasonably expected for impurities of few wt%.

### Magnetic characterization

**Figure 5:**
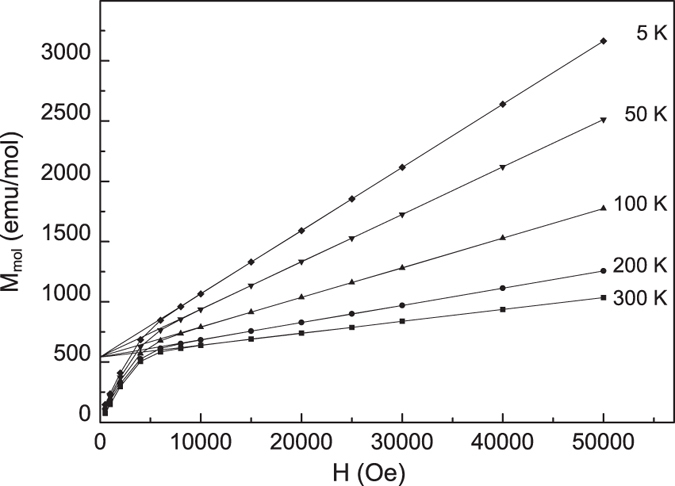
M vs H curves at different temperatures for the Fe02 sample. The linear fit of the high field region is also reported.

The magnetic measurements disclose a common behaviour for all the investigated samples. The temperature dependence of the magnetization (M(T)) evidences a paramagnetic (P) to antiferromagnetic (AF) transition with Néel temperature, T_N_, lower than 25 K, as generally observed for the lithium iron/manganese/cobalt orthosilicates^3^. Besides, the field dependence of the magnetization shows a non-linear behaviour at low magnetic fields in the whole investigated temperature range (5–300 K), revealing the presence of extrinsic ferromagnetic-like contributions, which also entail the M(T) curves to be shifted toward higher M values with respect to the usual Curie-Weiss behaviour expected for these orthosilicates^3^. In Fig. 5 the M vs H curves at different temperatures of the Fe02 sample are reported, as an example, together with the fit of the linear part of each curve.

**Figure 6:**
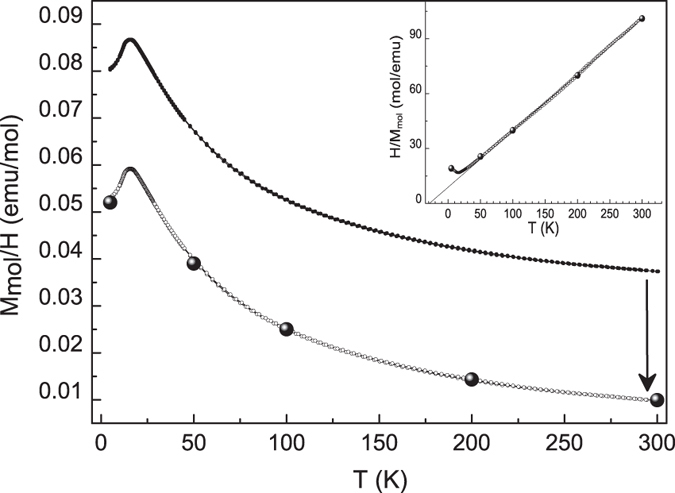
ZFC and FC temperature dependence of molar magnetization at 20000 Oe for Fe02 sample before (upper curve) and after (lower curve) subtracting the contribution of the saturated ferromagnetic impurities (see text). Symbols on the lower curve represent the χ values inferred from the slope of the linear fit of M vs H curves in the paramagnetic region (see Fig. 5). Inset: reciprocal of the lower M/H vs T curve and 1/χ values inferred by the M(H) linear fits.

As mentioned above, the non-linear M(H) behavior at low magnetic fields was observed at all the investigated temperatures and a value of magnetization at null field obtained by extrapolating the linear behavior at high magnetic fields, M_S_(H = 0), of about 540 emu/mol was inferred in the whole temperature range, corresponding to the residual magnetization of saturated ferromagnetic phases. For this sample, the presence of metallic cobalt is suggested by XRPD data and small quantities of magnetite are also evidenced by Mossbauer data (see the Supplementary Section). Taking into account the saturation magnetization values of these two ferromagnetic phases^37^,^38^, the expected contribution to the whole saturation magnetization of the sample indeed suitably matches the M_S_(H = 0) value obtained from the data reported in Fig. 5. A good agreement between the amount of ferromagnetic phases estimated on the basis of the M_S_(H = 0) values, and the respective amount reported in Table 1a,b has been similarly found for all the samples. Figure 6 reports the magnetization *vs* temperature behavior (M/H vs T curve) for the Fe02 sample (upper curve) evidencing the P-AF transition at about 16 K. The lower curve in the same figure has been obtained by subtracting the constant contribution coming from the ferromagnetic impurities in the sample, as determined from the curves reported in Fig. 5. Symbols on the lower curve represent the χ values inferred at the different temperatures from the slope of the linear part of the M vs H curves from Fig. 5, *i.e*. corresponding to the paramagnetic regime of the compound.

**Figure 7:**
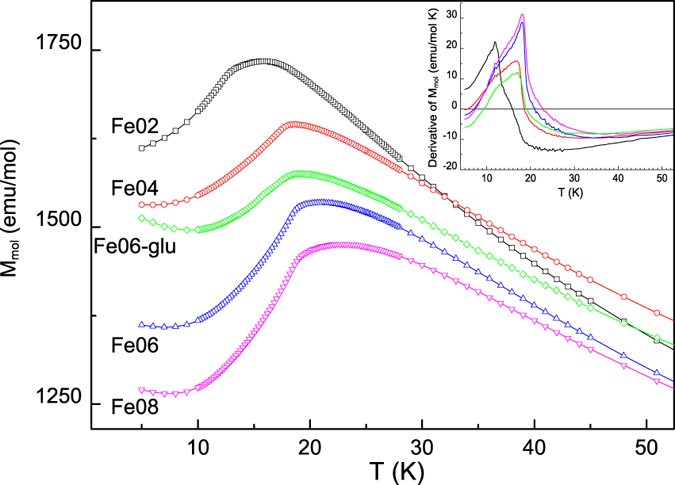
Temperature dependence of molar magnetization at 20000 Oe in the low temperature region for all the investigated samples. In the inset the M vs T derivative curves are reported for all the samples with the same color of the corresponding M vs T curve.

A very good agreement between the shifted M/H(T) curve and the χ values deriving by the M(H) linear fit is evident confirming that only saturated ferromagnetic phases are present together with the orthosilicate. In the inset of Fig. 6 the reciprocal of the shifted M/H vs T curve, together with the 1/χ values inferred by the M(H) linear fits, is reported. These data allowed us to estimate, for this sample, a Weiss-constant (θ) of about −29 K, in good agreement with the values typically found for these compounds^3^. Following the same procedure, a negative Weiss constant was determined for all the samples in the range 29 K < |θ| < 41 K, with |θ| values increasing at increasing the Fe content in the orthosilicate phase. In Fig. 7 the temperature dependence of the molar magnetization at 20000 Oe is reported (curves of Fe06, Fe08 and Fe06-glu, chosen as an example from the Fe-glu series, were translated for clarity of comparison) in the low temperature region, in order to evidence the presence of the P-AF transition in all the samples, with T_N_, ranging between 16 and 23 K, increasing at increasing of the Fe amount in the orthosilicate phase. In the inset of Fig. 7 the first derivatives of the M vs T curves are shown. Finally, for Li_2_CoSiO_4_ a T_N_ value of 18 K was obtained, in agreement with that reported in literature^3^,^39^.

### Cyclic voltammetry results

**Figure 8:**
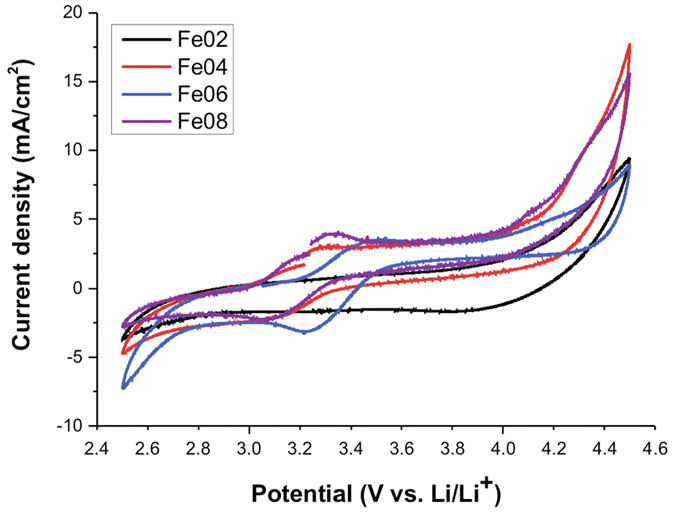
Cyclic voltammograms (2^nd^ cycle) for the mixed samples, recorded at 0.1 mV/sec between 2.5–4.5V.

Figure 8 shows the cyclic voltammograms (second cycle) for the Fe02, Fe04, Fe06 and Fe08 samples.

Intercalation and de-intercalation peaks can be clearly observed except for the Fe02 sample. These peaks are all in the range 3.2–3.5 V and can be easily ascribed to the Fe^2+^/Fe^3+^ redox couple. The second oxidation for iron is expected at voltages higher than 4.5 V, therefore beyond the voltage range in which we performed our measurements. The redox couples Mn^2+^/Mn^3+^ and Mn^3+^/Mn^4+^ for Li_2_MnSiO_4_ should have potential peaks around 4.1 and 4.5 V, according to the literature^28^, while for Li_2_CoSiO_4_ redox peaks are expected at about 4.2 and 5.0 V (Co^2+^/Co^3+^ and Co^3+^/Co^4+^)^28^. No obvious oxidation or reduction current can be observed in the CV curves for Mn or Co ions and the current density associated with the Fe^2+^/Fe^3+^ redox process can be related to the iron content in each sample. In fact, the Fe02 sample shows nearly a flat curve, while the peaks in the Fe06 and Fe08 CVs have a much higher current density. A shift in the peaks position towards higher voltage is observed for the Fe06 sample compared to Fe04 and Fe08. Since the *P2*_*1*_*/n* polymorph is the main phase for these samples, there are no evident structural differences which could explain this voltage shift. Anyway, we cannot neglect that the partial substitution of Fe with other cations in the crystalline structure can cause slightly different arrangements in the polyhedra, due to subtle differences in bond lengths which could clarify the shifts in the CV curves. Even so, a clear trend between shift and the composition of the silicate cannot be found immediately.

## Discussion and Conclusion

In our work we have shown that, although some optimization is still needed, the Li_2_Fe_1−x_Co_x/2_Mn_x/2_SiO_4_ (x = 0.2, 0.4, 0.6 and 0.8) samples were simply synthesized via solid state synthesis at an acceptable level of purity, as determined by the quantitative analysis performed by using the Rietveld method. For the actual application of these compounds as electrode materials in lithium cells, carbon coating is necessary due to their low intrinsic electronic conductivity (6 × 10^−14^ S cm^−1^). So, glucose was used as a source of carbon during the synthesis of some samples to check mainly if it could have any detrimental effect on the formation of the main phase. In fact, as reported previously^14^,^17^ carbon coating the β_II_ Li_2_CoSiO_4_ phase was not possible because of the formation of lithium silicate and Co metal. Electrodes made by using Li_2_CoSiO_4_ with no “internal” carbon showed a very poor performance^14^ thus reducing further possibility of employing this material in commercial Li cells. In general, in our case, we found that for the same composition, the glucose addition promoted the formation of impurity phases and lowered the crystallinity.

We determined the stable polymorphs for the different compositions thanks to a close inspection of XRPD data by applying the Rietveld structural refinement. Due to the similar scattering power of the three transition metals Fe, Co and Mn a precise determination of the cation ordering from XRPD data is not a trivial task. For this specific aim neutron diffraction data would be needed, but this is outside the aim of the present work. In both the series of samples, when iron ions were present in greater amount than Mn and Co the monoclinic *P2*_*1*_*/n* polymorph, *i. e*. the most stable polymorph for Li_2_FeSiO_4_, at least at this synthesis temperature^13^, was stabilized. In the case of the Fe02 sample, a mixture of polymorphs was instead observed, due to a clear preference of the transition metal ions for a specific crystal structure: Co ions guided the synthesis towards the formation of the *Pbn2*_*1*_ structure, while Fe and Mn segregated in the *Pmn2*_*1*_ polymorph. This sample also displayed peculiar magnetic features. The M vs T curve has a broad shape in the temperature region around the P-AF transition (see Fig. 7) with an even more marked difference in the first derivative of the same curve. The shape of this latter could reasonably come from the co-presence of phases characterized by two different T_N_ values, *i.e*. T_N_ ≈ 14 K, possibly due to the Mn/Fe orthosilicate, and T_N_ ≈ 18K, in agreement with the T_N_ value assigned to Li_2_CoSiO_4_ in this work. Then, the formation of a solid solution does not seem possible when the Co amount is higher than 0.3 atom in the silicate formula. The different behaviour between the two Fe02 samples must be obviously due to the carbon addition that could inhibit the reactivity of the mixture. For the Fe02-glu sample, Fe ions apparently did not easily enter in the silicate structures but segregated almost completely as magnetite phase. Therefore, the structures of the two stable polymorphs were those preferred by the Co ions: the *Pbn2*_*1*_ which could host mainly the Co ions, and the *Pmn2*_*1*_ crystal structure which instead, in this case, could host mainly the Mn ions, being frequently reported also for Li_2_MnSiO_4_. The sample is thus constituted by a mixture of the polymorphs typical of Co and Mn ions. XRPD and magnetic results thus suggest that an actual solid solution is possible only when either Fe, Mn and Co ions are equally present in the sample or when iron is the prevalent one.

The impurity phases were precisely identified and quantified by means of the Rietveld refinement (see Table 1a,b). The magnetic impurities, in particular Fe_3_O_4_, were also quantified by using Mössbauer and magnetization measurements and, in both cases, a good agreement with XRPD data was found. In addition, the combined use of Mössbauer and XRPD data allowed us to detect the presence, in the Fe06-glu sample, of a FeCo alloy, structurally similar to metallic iron. By using the amount of the secondary phases reported in Table 1a,b, the effective stoichiometry of the orthosilicate samples could be re-calculated: the formulas are reported in Table 4. Despite the precautions taken during the synthesis, a partial iron oxidation could not be avoided. By using the Mössbauer spectroscopy we were able to distinguish and quantify the Fe^3+^ ions belonging to the magnetite and orthosilicate phases: the results are reported in Table 4. The absolute percentage value of Fe^3+^ is quite constant (about 0.07–0.08 atoms in the formula, see Table 4). So, a sort of limit for the unavoidable oxidation process in the current experimental conditions (without glucose addition) can be suggested.

**Figure 9:**
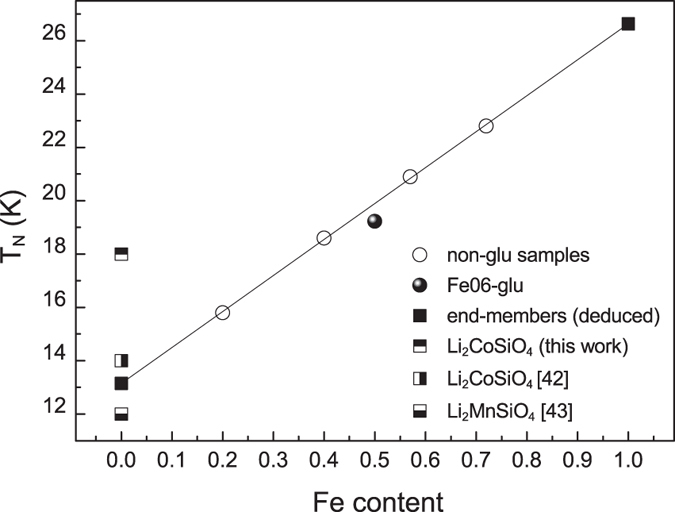
Néel temperatures (T_N_) as a function of the Fe amount in the orthosilicate phase for all the investigated samples and linear interpolation of the values for the mixed non-glu samples. The T_N_ values so deduced for the end-members (Fe = 0 and Mn,Co = 0) and the T_N_ values reported in literature for Li_2_CoSiO_4_ and Li_2_MnSiO_4_ are also included.

The presence of Fe^3+^, detected from Mössbauer measurements, is in agreement also with the values of the Néel temperatures inferred from the magnetic measurements. In our previous work^40^ a T_N_ value of 20 K was obtained for pure iron silicates prepared by sol-gel synthesis at 650 °C and 900 °C. This is not the case for the samples considered in this work, as shown in the following. A linear trend was obtained reporting in graph the T_N_ values as a function of the Fe amount in the orthosilicate phase, as shown in Fig. 9.

This trend is associable to the presence of antiferromagnetic super-exchange interactions stronger for higher Fe content, as also confirmed by the values of the Curie-Weiss constants, θ which similarly increases in absolute value by increasing the Fe amount, as the T_N_. The linear interpolation of the experimental T_N_ values allows us to predict, for a pure Li_2_FeSiO_4_, a T_N_ value of about 26K. This value is indeed compatible with the presence of Fe^3+^ ions on the Fe^2+^ sites. In fact, a T_N_ higher than 20 K for Li_2_FeSiO_4_ was already related to a consistent amount of Fe^3+^ in the orthosilicate phase by Mössbauer measurements performed at low temperature onto de-lithiated samples^41^. The authors reported a T_N_ = 20K for Li_2_FeSiO_4_, containing only Fe^2+^, and a T_N_ = 28 K for a de-lithiated sample with Li_1.34_Fe^2+^_0.33_Fe^3+^_0.66_SiO_4_ composition, suggesting that stronger AF interactions take place when Fe^2+^ ions are partially substituted by Fe^3+^ ions. The other end of the linear interpolation of the data of Fig. 9, corresponding to a Fe = 0 amount, is located at an intermediate position between the T_N_ values of pure Li_2_MnSiO_4_^40^ and Li_2_CoSiO_4_ this work and^39^  samples.

Moreover, some magnetic features are affected by the carbon coating. Looking at the linear behaviour in Fig. 9, a T_N_ value slightly lower than the expected one is disclosed for Fe06-glu, especially taking into account the re-calculated stoichiometry (Table 4) and, even more, considering the Fe^3+^ amount in the sample which should favour stronger AF local interactions and so a higher T_N_ value. The unexpected behaviour could be associated to the lower crystallinity degree of the sample induced by the use of glucose during the synthesis.

The cation distribution is hardly achievable from the Curie constant values, due to the presence of different kinds of cations. In any case, a fair agreement, above all for the samples with a low Fe amount, was found between the experimental and the expected C value, estimated according to the orthosilicate re-calculated stoichiometry and the Fe^2+^/Fe^3+^ amount in the sample (Table 4), taking into account the spin-only contribution of the cations to the average effective magnetic moment.

The CV curves were used to give an initial clue of the electrochemical behaviour of these mixed orthosilicates. Although promising, these orthosilicates often showed electrochemical performances well below the expectation and this is particularly true for Li_2_CoSiO_4_^14^. A first attempt has been done recently to investigate the performance of mixed Li_2_(FeMnCo)SiO_4_ compounds^27^ and a discouraging capacity fading was demonstrated after twenty cycles. In general, for all our samples, low current densities and broad redox peaks that disappeared after some cycles were observed, thus suggesting a poor electrochemical response for a future application. Although CV should be supported by more detailed investigations to draw clear conclusions, for example by using galvanostatic or potentiostatic intermitted titration technique GITT/PITT, our preliminary results showed only the redox reaction associated to the Fe^2+^/Fe^3+^ couple, thus revealing a scarce or absent electrochemical activity of the other cations in the considered voltage range. In addition, according to our Mössbauer results, a relevant percentage of Fe^3+^ was found in all the orthosilicates polymorphs that could have negatively affected the electrochemical response. It has also to be considered that a partially oxidized active material is likely to be sub-stoichiometric in lithium. These considerations could help in explaining the detected low peak current for all the samples. In particular the Fe02 sample did not show clearly any redox peaks; this was the sample with the lowest initial composition in iron and also with the highest amount of Fe^3+^ (about 40%). This means that just about half of the initial iron was available to be oxidized during the first charge, but actually no peaks, even of very low peak current, were observed either in the first or in the subsequent cycles. This could also suggest that Fe^3+^ in the crystalline structure cannot be electrochemically reduced to Fe^2+^ and then reversibly cycled.

In conclusion, Li_2_(FeCoMn)SiO_4_ samples in a wide compositional range with and without carbon were successfully synthesized by means of a simple and rapid solid state reaction. The formation of a stable solid solution with the preferred monoclinic *P2*_*1*_*/n* crystal structure was demonstrated when iron is prevalent in the initial composition or cobalt is below 0.3 atom per formula unit. In the other cases a mixture of *Pmn2*_*1*_ and *Pbn2*_*1*_ polymorphs, guided by cobalt and manganese ions is formed. The glucose addition during the synthesis increased the total amount of impurity phases. An unfavourable oxidation of iron ions in the present experimental conditions was found that was promoted by high Fe content, as demonstrated by Mössbauer spectroscopy. Accordingly, the electrochemical performances of these materials as cathodes in lithium-ion batteries are not yet fully optimized. Further investigation is needed to assess if cation mixing is truly beneficial for this system. Our findings suggest that the combined use of different techniques is currently a powerful tool for a thorough characterization of these samples. Work reported here can serve as a basis for the materials development oriented toward cathode materials.

## Methods

### Synthesis

The Li_2_Fe_1−x_Co_x/2_Mn_x/2_SiO_4_ (x = 0.2, 0.4, 0.6 and 0.8) samples were prepared by solid state synthesis. Stoichiometric amounts of Li_2_SiO_3_, CoCO_3_, FeC_2_O_4_·2H_2_O and MnCO_3_ were thoroughly mixed in a planetary miller by using tungsten carbide jars and spheres for total 6 hours at 600 rpm in multiple steps which included periods of rest. The same samples were also prepared with an additional grinding step of 30 min with 6 wt% of glucose addition. The obtained precursors were then treated in an oven, in N_2_ atmosphere at 700 °C for 10 hours. Li_2_CoSiO_4_ was also synthesized by using the same experimental procedure. In the following, the Li_2_Fe_0.2_Co_0.4_Mn_0.4_SiO_4_, Li_2_Fe_0.4_Co_0.3_Mn_0.3_SiO_4_, Li_2_Fe_0.6_Co_0.2_Mn_0.2_SiO_4_ and Li_2_Fe_0.8_Co_0.1_Mn_0.1_SiO_4_ samples will be named Fe02, Fe04, Fe06 and Fe08 respectively. The samples synthesized with the addition of glucose will be named Fe02-glu, Fe04-glu, Fe06-glu and Fe08-glu.

### Characterization techniques

X-ray powder diffraction (XRPD) measurements were performed by using a Bruker D5005 diffractometer with the CuK*α* radiation, graphite monochromator and scintillation detector. The patterns were collected in air with a step size of 0.02° and counting time of 10 s per step in the angular range 15–100°.

Rietveld structural and profile refinement was carried out by means of TOPAS V3.0 program^42^. During the refinement, in addition to the background, scale and zero error parameters, also the lattice parameters, isotropic thermal factors and atomic positions were allowed to vary. Due to the insignificant difference in the X-ray scattering power of Fe, Co and Mn ions, transition metal cations on the crystallographic sites were fixed at the stoichiometric values. The weight percentages of the impurity phases were also determined.

Room temperature Mössbauer spectra were collected by means of a proportional Kr-CO_2_ counter and a WissEl™ mod. MVT 1000 spectrometer, calibrated by using a standard metal iron foil, in the following velocity ranges depending on the impurities in the different samples:

- ±12 mm/s for Fe08 and Fe06;
- ±8 mm/s for Fe06-glu, Fe04 and Fe02.

The γ-ray source was a 25 mCi ^57^Co in Rhodium matrix with Lamb-Mössbauer factor *f*_s_ = 0.615, evaluated by applying the method described in ref. 43.

All the absorbers (14 mm diameter) were prepared to balance the signal to noise ratio and the distortion of the line shape due to saturation effect^44^. The obtained powder samples contained the following quantities of active material: 72 mg for Fe08, 119 mg for Fe06 and Fe06-glu, 143 mg for Fe04 and, finally, 180 mg for Fe02.

The aim of the present analysis is to reveal small site contribution ≤10% of the total iron amount. Since the standard fitting procedure based on Lorentzian profiles and linear approximation generally leads to erroneous evaluation of weak and/or poorly resolved contributions^45^,^46^, we chose to express the spectra line shape through the transmission integral function^44^ in order to take simultaneously all the broadening/distortion effects into account^47^.

The following expression


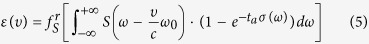


indicates the fractional intensity vs. Doppler velocity, where *f*_s_^r^ is the reduced source recoilless fraction defined and experimentally determined as in ref. 43 and 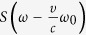 is the source line shape given by a Voigt profile whose Lorentzian component has natural line width and the Gaussian component is characterized by a standard deviation suitable to reproduce the total line width of the source as provided by the manufacturer (Γ_s_ = 1.03 mm/s). Moreover, *t*_a_ is the effective thickness number of the sample and, finally, *σ*(*ω*) is the Mössbauer cross-section, which depends on the relative contributions of the two iron oxidation states, Fe^2+^ and Fe^3+^, and on the presence of iron impurities.

The magnetic field dependence of magnetization, M(H), was investigated by means of a Quantum Design Squid magnetometer, at different temperatures with magnetic field ranging between 0 and 50000 Oe. M vs. T curves have been also collected in the range 5–300 K applying a 20000 Oe magnetic field, chosen in the field region where a linear M(H) dependence was observed for all the samples.

To prepare the cathode layer, a slurry was made by mixing the active materials with carbon black (Alfa) and poly(vinylidene fluoride) (PVdF, Solvay) in N-methyl-2-pyrrolidone (NMP, Aldrich) with a weight ratio of 70:20:10. The obtained suspensions were spread on an aluminium current collector by using a doctor blade. After the evaporation of the solvent in an oven at 80 °C overnight, the foils were transferred to an Ar filled dry-box (MBraun, <1 ppm O_2_, <1 ppm H_2_O). They were cut into disks of 1 cm diameter with a loading of about 4 mg/cm^2^ of active material. The electrochemical tests were performed using a three-electrodes T-cell with lithium metal as the counter and reference electrode, and a Whatman GF/A disc as the separator. The electrolyte was 1M LiPF_6_ in ethylene carbonate/diethyl carbonate (EC/DEC) 1:1 (Merck). All the cells were assembled in a dry-box under Argon atmosphere. The cyclic voltammetry (CV) was performed by using an Autolab PGSTAT30 (Metrohm) at a scan rate of 0.1 mV/s in the potential range 2.5–4.5 V. The cells were tested at room temperature (r.t.).

## Additional Information

**How to cite this article**: Ferrari, S. *et al*. New materials for Li-ion batteries: synthesis and spectroscopic characterization of Li_2_(FeMnCo)SiO_4_ cathode materials. *Sci. Rep*. **6**, 27896; doi: 10.1038/srep27896 (2016).

## Supplementary Material

Supplementary Information

## Figures and Tables

**Table 1:** Main structural parameters and weight percentages of orthosilicates polymorphs and impurity phases obtained from the Rietveld refinement for the samples (a) without glucose and (b) with glucose addition.

(a)						
	Fe08	Fe06	Fe04	Fe02		Li_2_CoSiO_4_
S.g.	*P2*_*1*_*/n*	P2_1_/n	*P2*_*1*_*/n*	*Pbn2*_*1*_	*Pmn2*_*1*_	*Pbn2*_*1*_
a (Å)	8.2441(3)	8.2392(2)	8.2457(6)	6.3443(16)	6.2568(4)	6.2718(2)
b (Å)	5.0136(1)	5.0131(1)	5.0086(2)	10.7326(25)	5.3741(4)	10.6897(3)
c (Å)	8.2299(3)	8.2503(2)	8.2531(5)	4.9983(8)	4.9542(3)	4.9302(1)
*β*°	98.91(1)	98.79(1)	98.70(3)			
V/Å^3^	336.06(1)	336.76(1)	336.93(3)	340.33(13)	166.58(2)	330.54(1)
R_wp_/S	9.60/1.33	9.72/1.36	10.09/1.46	9.77/1.43		10.38/1.32
Weight percentages						
Polymorph				21.08(48)	77.15(48)	
Fe_3_O_4_	5.93(9)	2.69(7)				
MnO_2_			1.35(17)			
Co		1.08(5)	2.99(8)	1.78(7)		2.3(1)
Li_2_Si_2_O_5_						4.1(2)
Li_2_SiO_3_		1.05(13)	0.92(21)			
**(b)**						
	**Fe08-glu**	**Fe06-glu**	**Fe04-glu**	**Fe02-glu**		
S.g.	*P2*_*1*_*/n*	*P2*_*1*_*/n*	*P2*_*1*_*/n*	*Pbn2*_*1*_	*Pmn2*_*1*_	
a (Å)	8.2283(6)	8.2407(5)	8.2415(10)	6.2690(14)	6.2861(5)	
b (Å)	5.0144(2)	5.0119(2)	5.0015(3)	10.7541(21)	5.3706(4)	
c (Å)	8.2473(5)	8.2544(6)	8.2557(8)	4.9599(7)	4.9465(4)	
*β*°	99.02(1)	98.87(1)	98.81(1)			
V/Å^3^	336.08(4)	336.83(3)	336.28(7)	334.38(11)	166.99(2)	
R_wp_/S	9.28/1.29	9.80/1.35	10.23/1.38	9.33/1.28		
Weight percentages						
Polymorph				41.1(5)	42.5(5)	
Fe	2.49(5)	5.14(7) FeCo				
Fe_3_O_4_	0.84(8)			10.10(15)		
MnO_2_			1.96(20)			
Co			4.42(8)			
Li_2_SiO_3_		3.13(21)	4.25(28)	6.30(23)		

The discrepancy factor and goodness of fit are also reported.

**Table 2:** Mössbauer fitting parameters for Fe08 (the isomer shift values are referred to standard iron at r.t.).

	Fe(II)			Fe(III)	Fe_3_O_4_	
	#1	#2	#3	#1	#1	#2
〈δ〉_*i*_	0.961(2)	0.963(3)	1.00(4)	0.223(9)	0.276(5)	0.66(1)
〈Δ〉_*i*_	2.41(1)	2.46(1)	2.27(7)	0.93(2)	−0.02(1)	−0.03(2)
〈*B*〉_*i*_					49.18(3)	46.17(7)
*t*_*i*_	2.4(4)	2.2(4)	0.30(3)	0.53(2)	0.37(1)	0.53(2)
σ_*ι*_	≈0	0.048(6)	0.46(4)	0.193(7)	0.068(7)	0.11(2)
σ_*ι*_^(*B*)^					≈0	1.4(1)

**Table 3:** Mössbauer fitting parameters for Fe06-glu (the isomer shift values are referred to standard iron at r.t.).

	Fe(II)			Fe(III)	FeCo	
	#1	#2	#3	#1	#1	#2
〈δ〉_*i*_	0.965(1)	0.990(4)	0.9(2)	0.24(1)	0.012(7)	0.5(2)
〈Δ〉_*i*_	2.445(1)	2.74(2)	2.2(5)	0.96(2)	0.02(1)	≈1.5
〈*B*〉_*i*_					32.57(5)	≈3
*t*_*i*_	5.90(8)	0.42(6)	0.2(1)	1.62(4)	0.26(1)	0.3(1)
σ_*ι*_	≈0	0.04(1)	0.3(1)	0.174(4)	0.06(2)	≈0
σ_*ι*_^(*B*)^					0.7(1)	≈10

**Table 4:** Re-calculated stoichiometry (see text) and absolute and percentage of Fe^2+^ and Fe^3+^ in the orthosilicate phase.

Sample	Orthosilicate re-calculated stoichiometry	Fe^2+^ and Fe^3+^ amount in the formula	Fe^2+^ and Fe^3+^ % on the total orthosilicate amount
Fe08	Li_2_Fe_0.72_Co_0.1_Mn_0.1_SiO_4_	Fe^2+^_0.653_Fe^3+^_0.067_	Fe^2+^ 90.7 Fe^3+^ 9.3
Fe06	Li_2_Fe_0.57_Co_0.17_Mn_0.2_SiO_4_	Fe^2+^_0.489_Fe^3+^_0.081_	Fe^2+^ 85.7 Fe^3+^ 14.3
Fe06-glu	Li_2_Fe_0.5_Co_0.2_Mn_0.2_SiO_4_	Fe^2+^_0.399_Fe^3+^_0.101_	Fe^2+^ 79.8 Fe^3+^ 20.2
Fe04	Li_2_Fe_0.4_Co_0.23_Mn_0.29_SiO_4_	Fe^2+^_0.324_Fe^3+^_0.076_	Fe^2+^ 81.0 Fe^3+^ 19.0
Fe02	Li_2_Fe_0.2_Co_0.36_Mn_0.4_SiO_4_	Fe^2+^_0.116_Fe^3+^_0.084_	Fe^2+^ 58.0 Fe^3+^ 42.0

## References

[b1] Nyten A., Abouimrane A., Armand M., Gustafsson T. & Thomas J. O. Electrochemical performance of Li_2_FeSiO_4_ as a new Li-battery cathode material. Electrochem. Commun. 7, 156–160 (2005).

[b2] Dominko R. . Structure and electrochemical performance of Li_2_MnSiO_4_ and Li_2_FeSiO_4_ as potential Li-battery cathode materials. Electrochem. Comm. 8, 217–222 (2006).

[b3] Gong Z. & Yang Y. Recent advances in the research of polyanion-type cathode materials for Li-ion batteries. Energy Environ. Sci. 4, 3223–3242 (2011).

[b4] Guo H. . Optimum synthesis of Li_2_Fe_1−*x*_Mn_*x*_SiO_4_/C cathode for lithium ion batteries. Electrochimica Acta 55, 8036–8042 (2010).

[b5] Dominko R. . On the Origin of the Electrochemical Capacity of Li_2_Fe_0.8_Mn_0.2_SiO_4_ Batteries and Energy Storage. J. Electrochem. Soc. 157, A1309–A1316 (2010).

[b6] Islam M. S. . Silicate cathodes for lithium batteries: alternatives to phosphates? J. Mater. Chem. 21, 9811–9818 (2011).

[b7] Zhang S., Deng C., Fu B. L., Yang S. Y. & Ma L. Effects of Cr doping on the electrochemical properties of Li_2_FeSiO_4_ cathode material for lithium-ion batteries. Electrochimica Acta 55, 8482–8489 (2010).

[b8] Li Y., Cheng X. & Zhang Y. Achieving High Capacity by Vanadium Substitution into Li_2_FeSiO_4_. J. Electrochem. Soc. 159, A69–A74 (2012).

[b9] Zhang S., Deng C., Fu B. L., Yang S. Y. & Ma L. Doping effects of magnesium on the electrochemical performance of Li_2_FeSiO_4_ for lithium ion batteries. J. Electroanalytical Chemistry 644, 150–154 (2010).

[b10] Deng C., Zhang S., Yang S. Y., Fu B. L. & Ma L. Synthesis and characterization of Li_2_Fe_0.97_M_0.03_SiO_4_ (M = Zn^2+^, Cu^2+^, Ni^2+^) cathode materials for lithium ion batteries. J. Power Sources 196, 386–392 (2011).

[b11] Moskon J., Dominko R., Cerc-Korosec R., Gaberscek M. & Jamnik J. Morphology and electrical properties of conductive carbon coatings for cathode materials. J. Power Sources 174, 683–688 (2007).

[b12] Huang X. . Synthesis and electrochemical performance of Li_2_FeSiO_4_/carbon/carbon nano-tubes for lithium ion battery. Electrochimica Acta 55, 7362–7366 (2010).

[b13] Sirisopanaporn C. . Polymorphism in Li_2_(Fe,Mn)SiO_4_: A combined diffraction and NMR study. J. Mater. Chem. 21, 17823–17831 (2011).

[b14] He G., Popov G. & Nazar L. F. Hydrothermal Synthesis and Electrochemical Properties of Li_2_CoSiO_4_/C Nanospheres. Chem. Mater. 25, 1024−1031 (2013).

[b15] Mali G., Sirisopanaporn C., Masquelier C., Hanzel D. & Dominko R. Li_2_FeSiO_4_ Polymorphs Probed by ^6^Li MAS NMR and ^57^Fe Mössbauer Spectroscopy. Chem. Mater. 23, 2735–2744 (2011).

[b16] Mali G., Meden A. & Dominko R. ^6^Li MAS NMR spectroscopy and first-principles calculations as a combined tool for the investigation of Li_2_MnSiO_4_ polymorphs. Chem. Comm. 46, 3306–3308 (2010). 20372695 10.1039/c003065a

[b17] Lyness C., Delobel B., Armstrong A. R. & Bruce P. G. The lithium intercalation compound Li_2_CoSiO_4_ and its behaviour as a positive electrode for lithium batteries. Chem. Comm. 4890–4892 (2007). 18361359 10.1039/b711552k

[b18] Lee H. . Origin of Poor Cyclability in Li_2_MnSiO_4_ from First-Principles Calculations: Layer Exfoliation and Unstable Cycled Structure. Chem. Mater. 26, 3896–3899 (2014).

[b19] Ferrari S. . Electrochemistry of orthosilicate-based lithium battery cathodes: a perspective. Phys. Chem. Chem. Phys. 16, 10353–10366 (2014). 24764049 10.1039/c4cp00511b

[b20] Dominko R. Li2MSiO4 (M = Fe and/or Mn) cathode materials. J. Power Sources 184, 462–468 (2008).

[b21] Kokalj A. . Beyond One-Electron Reaction in Li Cathode Materials: Designing Li_2_Mn_*x*_Fe_1−*x*_SiO_4_. Chem Mater. 19, 3633–3640 (2007).

[b22] Gong Z. L., Li Y. X. & Yang Y. Synthesis and Characterization of Li_2_Mn_*x*_ Fe_1−*x*_ SiO_4_ as a Cathode Material for Lithium-Ion Batteries, Fuel Cells, and Energy Conversion. Electrochem. Solid-State Lett. 9, A542–A544 (2006).

[b23] Kim J. C., Li X., Kang B. & Ceder G. High-rate performance of a mixed olivine cathode with off-stoichiometric composition. Chem. Comm. 51, 13279–13282 (2015). 26199196 10.1039/c5cc04434k

[b24] Kim J. C. . Analysis of Charged State Stability for Monoclinic LiMnBO_3_ Cathode. Chem. Mater. 26, 4200–4206 (2014).

[b25] Kim J. C., Seo D.-H. & Ceder G. Theoretical capacity achieved in a LiMn_0.5_Fe_0.4_Mg_0.1_BO_3_ cathode by using topological disorder. Energy Environ. Sci. 8, 1790–1798 (2015).

[b26] Nitta N., Wu F., Tae Lee J. & Yushin G. Li-ion battery materials: present and future. Materials Today 18, 252–264 (2015).

[b27] Yamashita H., Ogami T. & Kanamura K. Hydrothermal Synthesis and Electrochemical Properties of Li_2_Fe_x_Mn_x_Co_1−2x_SiO_4_/C Cathode Materials for Lithium-ion batteries. Electrochemistry 83, 413–420 (2015).

[b28] Dompablo M. E. A., Armand M., Tarascon J. M. & Amador U. On-demand design of polyoxianionic cathode materials based on electronegativity correlations: An exploration of the Li_2_MSiO_4_system (M = Fe, Mn, Co, Ni). Electrochem. Comm. 8, 1292–1298 (2006).

[b29] Shannon R. D. Revised Effective Ionic Radii and Systematic Studies of Interatomic Distances in Halides and Chaleogenides. Acta Cryst. A32, 751–767 (1976).

[b30] Rancourt D. G. In: Mössbauer Spectroscopy Applied to Magnetism and Materials Science, vol. 2, (eds Long G., Grandjean F.), (Plenum Press, 1996).

[b31] Wu X., Jiang X., Huo Q. & Zhang Y. Facile synthesis of Li_2_FeSiO_4_/C composites with triblock copolymer P123 and their application as cathode materials for lithium ion batteries. Electrochimica Acta 80, 50–55 (2012).

[b32] Janot C. L.’effet Mössbauer et ses applications, (Masson et C. ie, 1972).

[b33] Bancroft G. M. Mössbauer Spectroscopy, (McGraw Hill, 1973).

[b34] Zhou D., Li Z. W., Yang X., Wen F. S. & Li F. S. Fabrication and Mössbauer Study of FeCo Alloy Nanotube Array. Chinese Physics Letters 25, 1865–1867 (2008).

[b35] Petrov Y. I. & Shafranovskii E. A. Specific features of the hyperfine field at iron nuclei in aerosol nanoparticles of FeCo alloy. Doklady Physical Chemistry 440, 178–182 (2011).

[b36] Kodama D. . Chemical Synthesis of Sub-micrometer- to Nanometer-Sized Magnetic FeCo. Advanced Materials 18, 3154–3159 (2006).

[b37] Azzoni C. B., Mozzati M. C., Massarotti V., Capsoni D. & Bini M. New insights into the magnetic properties of the Ca_2_Fe_2_O_5_ ferrite. Solid State Sciences 9, 515–520 (2007).

[b38] Kittel C. Introduction to Solid State Physics, second edition, (John Wiley & Sons, 1965).

[b39] Avdeev M., Mohamed Z. & Ling C. D. Magnetic structures of βI-Li_2_CoSiO_4_ and γ0-Li_2_MnSiO_4_: Crystal structure type vs. magnetic topology. J. Solid State Chem. 216, 42–48 (2014).

[b40] Bini M. . Polymorphism and magnetic properties of Li_2_MSiO_4_ (M = Fe, Mn) cathode materials. Sci. Rep. 3, 3452 (2013). 24316682 10.1038/srep03452 PMC6506446

[b41] Lee I. K., Kim S. J., Kouh T. & Kim C. S. Mössbauer analysis of silicate Li_2_FeSiO_4_ and delithiated Li_2−x_FeSiO_4_ (x = 0.66) Compounds. J. Appl. Phys. 113, 17E306–17E306-3 (2013).

[b42] Bruker AXS. *TOPAS V3.0: General profile and structural analysis software for powder diffraction data* (2005).

[b43] Spina G. & Lantieri M. A straightforward experimental method to evaluate the Lamb-Mössbauer factor of ^57^Co/Rh source. Nucl. Instr. and Meth. 218, 253–257 (2014).

[b44] Yi-Long C. & De-Ping Y. Mössbauer Effect in Lattice Dynamics, (Wiley-VCH Verlag GmbH & Co., 2007).

[b45] Bancroft G. M. Mössbauer spectroscopy: an introduction for Inorganic Chemists and Geochemists ; (McGraw Hill, 1973).

[b46] Bini M. . Pair distribution function analysis and Mossbauer study of defects in microwave-hydrothermal LiFePO_4_. RSC Advances 2, 250–258 (2012).

[b47] Rancourt D. G. Accurate site populations from Mössbauer spectroscopy. Nucl. Instr. and Meth. B44, 199–210 (1989).

