# Supplementary material for: New materials for Li-ion batteries: synthesis and spectroscopic characterization of Li2(FeMnCo)SiO4 cathode materials
Source: Sci Rep. 2016 Jun 13;6:27896. doi: 10.1038/srep27896 (PMC4904220; doi:10.1038/srep27896)

New materials for Li-ion batteries: synthesis and spectroscopic characterization of Li_2_(FeMnCo)SiO_4_ cathode materials

Stefania Ferrari^a^, Maria Cristina Mozzati^b^, Marco Lantieri^c^, Gabriele Spina^d^, Doretta Capsoni^e^ and Marcella Bini^e,*^

**Detailed analysis of the Mossbauer spectra**

Fe06

The Mössbauer cross section was expressed by means of six contributions: three of them belonging to Fe(II) sites, one to Fe(III) and, finally, two to magnetic impurities. The parameters of each contribution are reported in Table 1 and the cross sections due to Fe(II) sites (78%), Fe(III) site (13%) and impurities sites (9%) are illustrated in Figure 1 (left side). With reference to *σ_i_* values, the first contribution of Fe(II) can be associated to well crystalline regions in the sample, the second one to regions of medium degree of crystallinity and the last one (with *σ_i_* ≈ 2) to very poorly crystalline regions. The contribution of Fe(III) is connected with medium crystalline regions. The kind of the impurity phase, which is magnetite, was determined on the basis of the hyperfine parameters of the corresponding sites and its relative amount, which was calculated using equation (1) (see text), is *α* ≈ 0.026, which confirms the XRPD and Rietveld results, too.

Fe04

The Mössbauer cross section was expressed by means of four contributions: three of them belonging to Fe(II) sites and one to Fe(III). No significant evidence of magnetic impurities was observed. In Table 2 we report the parameters of each contribution and in Figure 2 (left side) we illustrate the cross sections due to Fe(II) sites (81%) and Fe(III) site (19%). Regarding the crystallinity, the *σ_i_* values reveal very well crystalline regions for the first contribution of Fe(II) accompanied by medium and very poorly crystalline regions for the other two contributions, in agreement with the significantly higher peak broadening of the XRPD patterns. The contribution of Fe(III) is connected with medium crystalline regions.

Fe02

In order to reproduce the experimental line shape, we expressed the Mössbauer cross section by means of six contributions: three of them belonging to Fe(II) sites, two to Fe(III) and, finally, one to a very weak magnetic impurity. In Table 3 we report the parameters of each contribution and in Figure 2 (right side) we illustrate the cross sections due to Fe(II) sites (57%), Fe(III) sites (41%) and impurity site (2%). With reference to *σ_i_* values, the first contribution of Fe(II) can be associated to medium crystalline regions, the second one to well crystalline regions and the last one to poorly crystalline regions. The first contribution of Fe(III) is connected with medium crystalline regions while the second one to poorly crystalline regions. We underline that for this sample the poorly crystalline regions are predominant, in agreement with the significantly higher peak broadening of the XRPD patterns. The kind of the impurity phase, which is magnetite, was determined on the basis of the hyperfine parameters of the corresponding site and its relative amount, which was calculated using equation (1), is *α* ≈ 0.002. This very small value confirms the fact that XRPD did not reveal any evidence of impurity.

Table 1: Mössbauer fitting parameters for Fe06 (the isomer shift values are referred to standard iron at r.t.)

|  | Fe(II) | | | Fe(III) | Fe_3_O_4_ | |
| --- | --- | --- | --- | --- | --- | --- |
|  | #1 | #2 | #3 | #1 | #1 | #2 |
|  | 0.963(1) | 0.97(1) | 0.8(1) | 0.24(1) | 0.266(8) | 0.66(2) |
|  | 2.449(1) | 2.50(3) | ≈ 0 | 0.97(3) | 0.00(2) | -0.04(4) |
|  |  |  |  |  | 49.21(6) | 46.2(2) |
| *t_i_* | 3.66(8) | 0.72(8) | 0.25(2) | 0.76(2) | 0.21(1) | 0.30(2) |
| *σ_ι_* | 0.026(3) | 0.14(1) | ≈ 2 | 0.17(1) | 0.06(3) | 0.26(6) |
| *σ_ι_*(*^B^*) |  |  |  |  | 0.5(2) | 1.6(4) |

Table 2: Mössbauer fitting parameters for Fe04 (the isomer shift values are referred to standard iron at r.t.)

|  | Fe(II) | | | Fe(III) |
| --- | --- | --- | --- | --- |
|  | #1 | #2 | #3 | #1 |
|  | 0.964(1) | 0.976(3) | 0.96(8) | 0.214(5) |
|  | 2.463(2) | 2.542(7) | 2.4(1) | 0.91(1) |
|  |  |  |  |  |
| *t_i_* | 2.89(8) | 1.83(7) | 0.21(2) | 1.14(1) |
| *σ_ι_* | ≈ 0 | 0.107(3) | 0.8(1) | 0.184(3) |
| *σ_ι_*(*^B^*) |  |  |  |  |

Table 3: Mössbauer fitting parameters for Fe02 (the isomer shift values are referred to standard iron at r.t.)

|  | Fe(II) | | | Fe(III) | | Fe_3_O_4_ |
| --- | --- | --- | --- | --- | --- | --- |
|  | #1 | #2 | #3 | #1 | #2 | #1 |
|  | 0.971(3) | 0.990(5) | 0.99(9) | 0.199(4) | 0.54(8) | 0.36(4) |
|  | 2.75(2) | 2.86(2) | 2.7(2) | 0.754(8) | 0.7(1) | -0.23(8) |
|  |  |  |  |  |  | 48.7(2) |
| *t_i_* | 1.5(2) | 0.6(2) | 0.25(7) | 1.2(1) | 0.5(1) | 0.07(2) |
| *σ_ι_* | 0.075(5) | ≈ 0 | 0.4(1) | 0.13(1) | 0.4(2) | ≈ 0.1 |
| *σ_ι_*(*^B^*) |  |  |  |  |  | ≈ 1 |

Figure 1 – Mössbauer cross section line shapes for the three sites of Fe06 illustrated in semi-logarithmic scale in order to better display the contributions due to Fe(III) (blue plot) and to impurities (green plot) with respect to the main one belonging to Fe(II) (red plot).


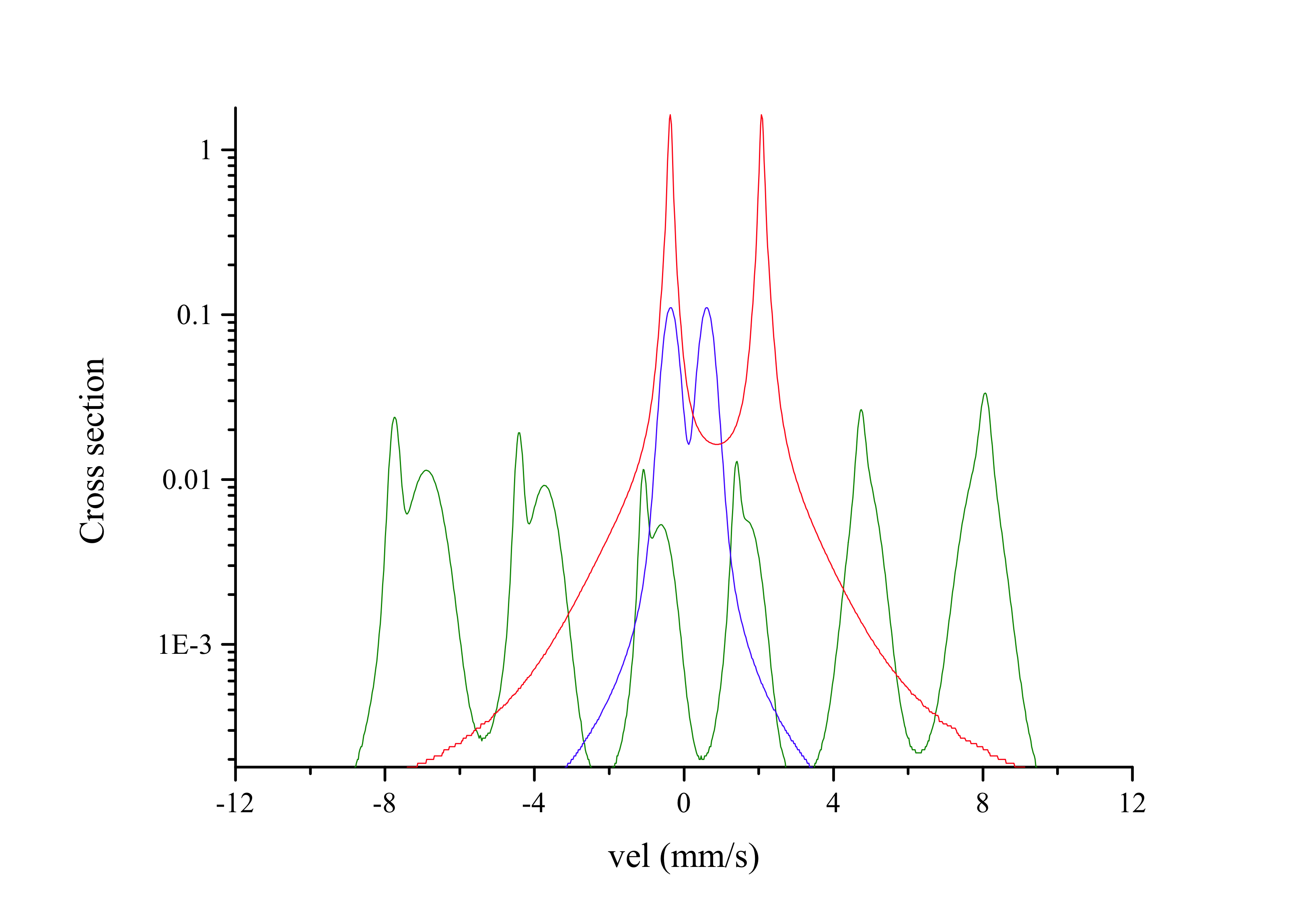


Figure 2 – Mössbauer cross section line shapes for the sites of Fe04 (left) and Fe02 (right) illustrated in semi-logarithmic scale in order to better display the contributions due to Fe(III) (blue plot) and to impurities (green plot) with respect to the main one belonging to Fe(II) (red plot).


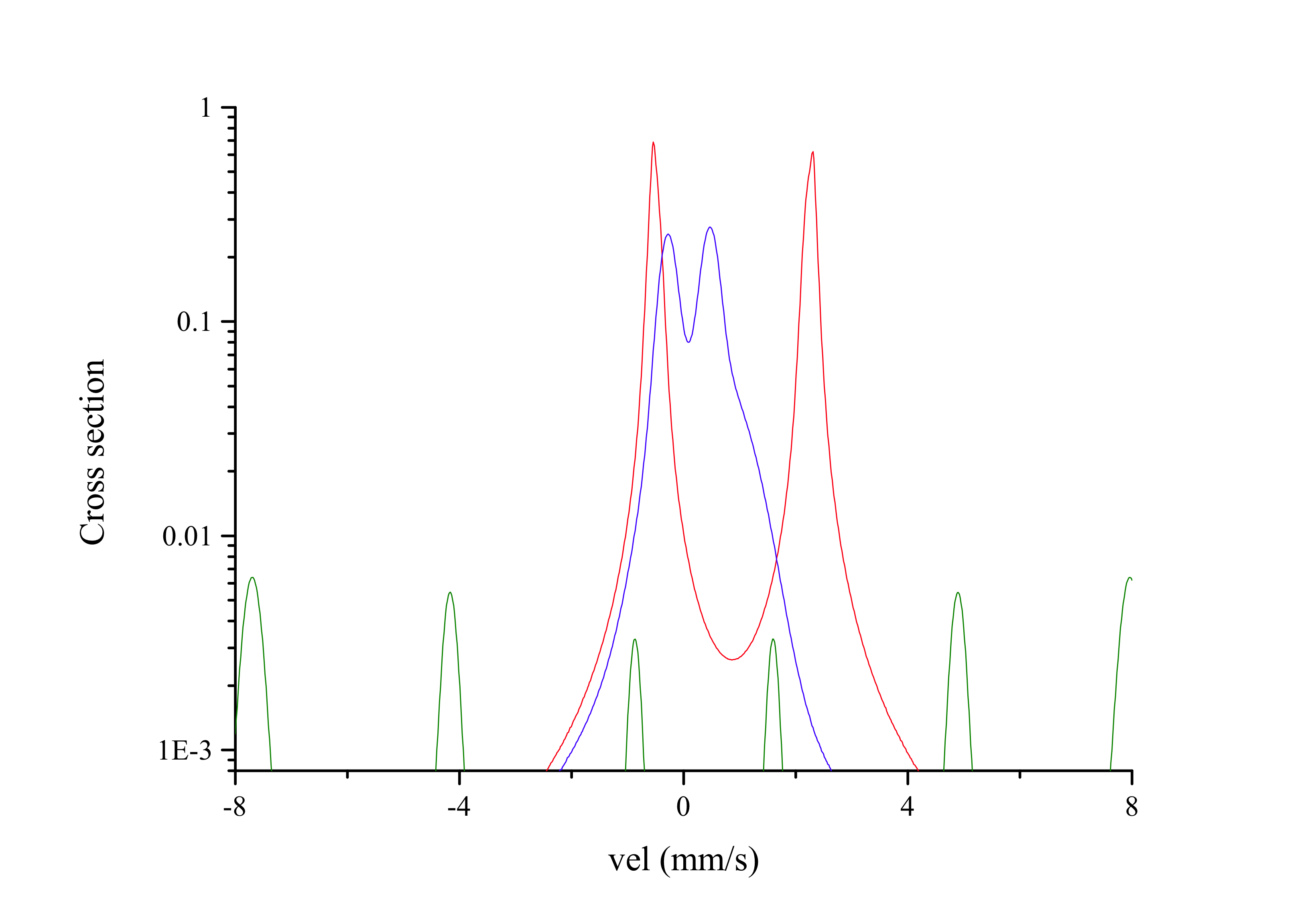

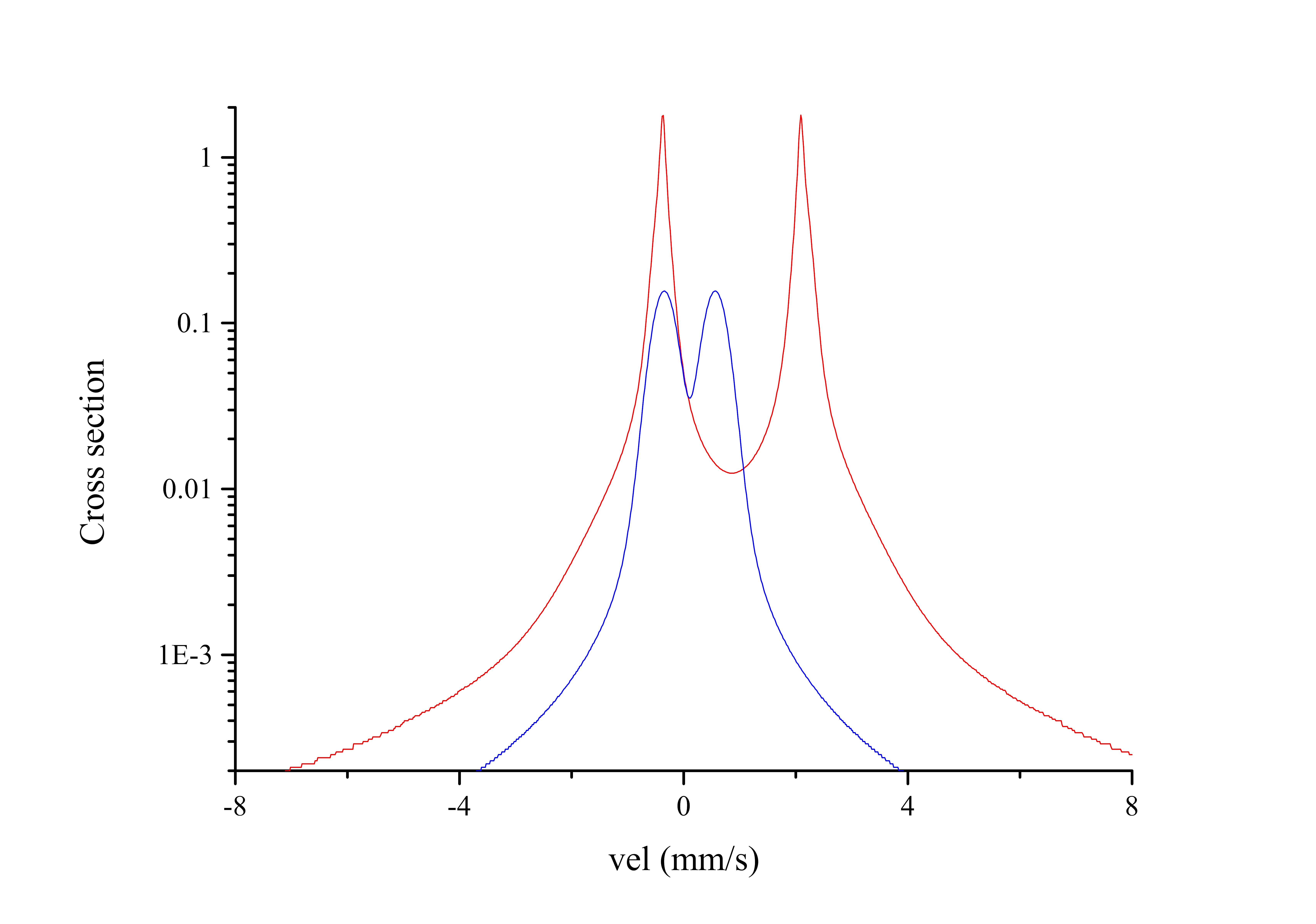

Supplement: Supplementary Information [file srep27896-s1.doc]
